# Cyanoacrylate pulmonary embolism after endoscopic sclerotherapy of gastric varices

**DOI:** 10.31744/einstein_journal/2021AI5778

**Published:** 2021-01-22

**Authors:** Julliana dos Santos Frassei, Camila Soares Franco, Vinicius Roeffero Brambilla, Bruna Melo Coelho Loureiro, Carolina dos Santos Kiebert, Eduardo Kaiser Ururahy Nunes Fonseca, Sabrina de Mello Ando, Marcio Valente Yamada Sawamura

**Affiliations:** 1 Hospital das Clínicas Faculdade de Medicina Universidade de São Paulo São PauloSP Brazil Hospital das Clínicas , Faculdade de Medicina , Universidade de São Paulo , São Paulo , SP , Brazil .

**Figure 1:**
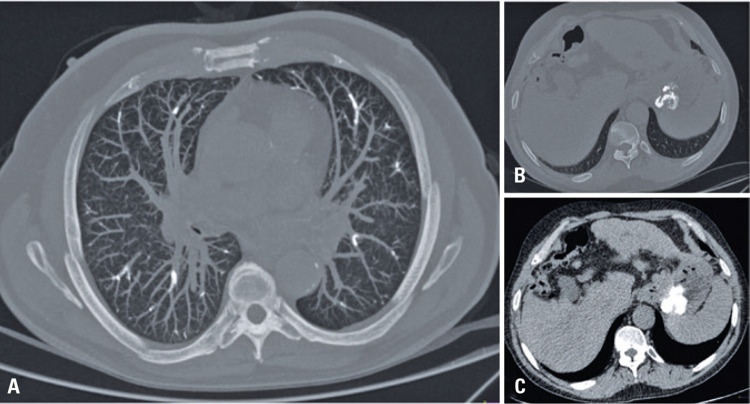
Tomography without contrast agents in axial cut of the chest (A) showing filling of the lung arteries by hyperattenuating material (cyanoacrylates solution and Lipiodol ® ). This is also the material of the same attenuation in the topography of the stomach in the bone and abdomen window (B), where previously gastric fundal varices were characterized (C)

**Figure 2 f02:**
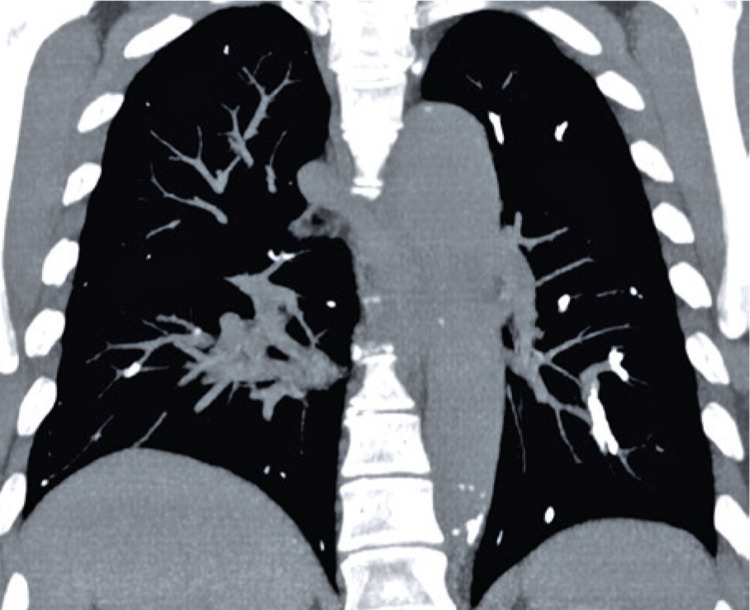
Tomography without contrast in the coronal section, in the mediastinum window the filling of multiple pulmonary arteries is shown by hyperattenuating material (cyanoacrylate solution and Lipiodol ® )

**Figure 3 f03:**
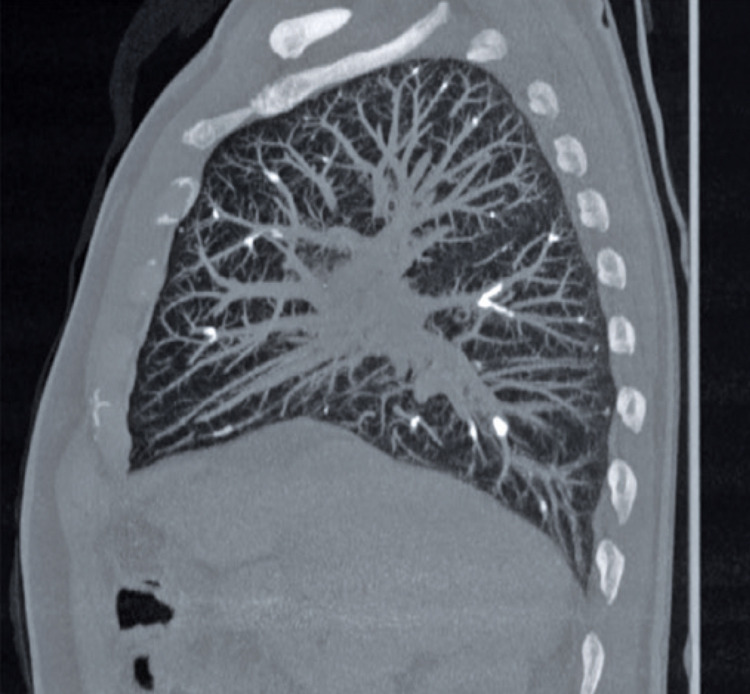
Tomography without contrast in sagittal section, in the mediastinum window, showing the same findings

This was a 65-year-old male patient with cirrhosis who experienced sudden dyspnea and thoracic pain few hours after underwent endoscopic sclerotherapy of gastric and esophageal varices by cyanoacrylate solution injection and Lipiodol ^®^ . The patient underwent computed tomography of the chest (Figures 1 to 3 [Fig f02] [Fig f03]).

Endoscopic treatment of gastric varices with cyanoacrylate is efficient to control bleeding in patients with liver disease that often results in the immediate hemostasis and varices caliber decrease in the first administration. ^( 1 , 2 )^ However, the procedure is not free of risk: among complications observed in the procedure, the embolization of polymers through lung arteries, coronary, cerebral and other vessels have been described. ^( 3 - 6 )^

Understating physiopathology of pulmonary embolism by such viewing overcome the fact that cyanoacrylate is a liquid monomers that is polymerized rapidly when in contact with blood, and may result in blood clotting and almost immediate vascular occlusion. To avoid the risk of the injecting needle to attach to the vessel and cause a severe bleeding when removed, the use of Lipiodol ^®^ is often associated with cyanoacrylate injection. As a result of this mixture, there is a delay in the polymerization process and in the cyanoacrylate clotting, therefore increasing the risk to embolization in the procedure. ^( 4 , 5 )^

The real incidence of this complication is unknown, once the assessment by imaging exam is not always conducted. The risk is believed to be higher to patients with extensive varices, which require higher volumes of sclerosing agents. There is also an association with the proportion of other components, such as the Lipiodol ^®^ . ^( 5 )^

Embolic complications due to the use of cyanoacrylate seems to be infrequent and they present a wide spectrum of symptoms, often without evolve with important morbimortality for patients. This is a safety and efficient procedure for the proposed goal. In this case report, the patient was referred to the emergency unit and received supportive measures and remained under surveillance for 12 hours, being discharged with good health status and asymptomatic.
