# Supplementary material for: Cyanoacrylate pulmonary embolism after endoscopic sclerotherapy of gastric varices
Source: Einstein (Sao Paulo). 2021 Jan 22;19:eAI5778. doi: 10.31744/einstein_journal/2021AI5778 (PMC7821951; doi:10.31744/einstein_journal/2021AI5778)
Supplement: Translation

# Translation: Embolização pulmonar por cianoacrilato após obliteração de varizes gástricas

**Authors:** Julliana dos Santos Frassei, Camila Soares Franco, Vinicius Roeffero Brambilla, Bruna Melo Coelho Loureiro, Carolina dos Santos Kiebert, Eduardo Kaiser Ururahy Nunes Fonseca, Sabrina de Mello Ando, Marcio Valente Yamada Sawamura

**Affiliations:** 1 Brasil Hospital das Clínicas, Faculdade de Medicina, Universidade de São Paulo, São Paulo, SP, Brasil .

Paciente do sexo masculino de 65 anos, com cirrose hepática, apresentou dispneia súbita e dor torácica algumas horas após ser submetido à obliteração de varizes gástricas e esofágicas por meio de injeção de solução de cianoacrilato e Lipiodol ^®^ . Foi submetido à tomografia computadorizada do tórax (Figuras 1 a 3 [Fig f02002] [Fig f03002]).

**Figura 1:** Tomografia sem contraste em corte axial do tórax (A) evidencia preenchimento de artérias pulmonares por material hiperatenuante (solução de cianoacrilato e Lipiodol ® ). Nota-se também material de mesma atenuação na topografia do estômago na janela óssea (B) e de abdome, onde previamente se caracterizavam varizes de fundo gástrico (C)

**Figura 2 f02002:** Tomografia sem contraste no corte coronal, na janela de mediastino evidencia o preenchimento de múltiplas artérias pulmonares por material hiperatenuante (solução de cianoacrilato e Lipiodol ® )

**Figura 3 f03002:** Tomografia sem contraste no corte sagital, na janela de mediastino, mostrando os mesmos achados

O tratamento endoscópico de varizes gástricas com cianoacrilato é eficaz no controle de sangramentos em pacientes hepatopatas, geralmente resultando na hemostasia imediata e diminuição do calibre das varizes na primeira aplicação. ^( 1 , 2 )^ No entanto, não é isento de riscos: dentre as complicações inerentes ao procedimento, a embolização do polímero resultante para artérias pulmonares, coronárias, cerebrais e outros vasos tem sido descrita. ^( 3 - 6 )^

O entendimento da fisiopatologia da embolização pulmonar por tal via perpassa o fato do cianoacrilato ser um monômero líquido que se polimeriza rapidamente quando em contato com o sangue, resultando em coagulação sanguínea e oclusão vascular quase imediata. Para evitar o risco de a agulha injetora aderir ao vaso e causar um sangramento grave ao ser retirada, geralmente o Lipiodol ^®^ é associado à injeção de cianoacrilato. Como resultado dessa mistura, ocorre um atraso no processo de polimerização e coagulação do cianoacrilato, aumentando o risco de embolização do procedimento. ^( 4 , 5 )^

A incidência real dessa complicação não é conhecida, uma vez que a avaliação por exame de imagem não é sempre realizada. Acredita-se que o risco pode ser maior em pacientes com varizes extensas, que exigem maiores volumes de materiais esclerosantes. Associa-se, também, com a proporção dos outros constituintes, como o Lipiodol ^®^ . ^( 5 )^

As complicações embólicas secundárias ao uso de cianoacrilato parecem ser infrequentes e apresentam um diverso espectro de sintomas, geralmente sem cursar com importante morbimortalidade para o paciente, sendo um procedimento seguro e eficaz para o objetivo proposto. No caso apresentado, o paciente foi encaminhado ao pronto atendimento do serviço, recebeu medidas de suporte e ficou em observação por 12 horas, recebendo alta estável e assintomático.
